# Safety and effectiveness of high flow extracranial to intracranial saphenous vein bypass grafting in the treatment of complex intracranial aneurysms: a single-centre long-term retrospective study

**DOI:** 10.1186/s12883-021-02339-w

**Published:** 2021-08-09

**Authors:** Jibo Zhang, Yu Feng, Wenyuan Zhao, Kui Liu, Jincao Chen

**Affiliations:** 1 Department of Neurosurgery, Zhongnan Hospital of Wuhan University, Donghu Road 169, Wuhan, 430071 China; 2 Department of Neurologic Surgery, Mayo Clinic, Rochester, MN 55905 USA

**Keywords:** High flow bypass, Extracranial to intracranial, Saphenous vein, Complex intracranial aneurysms

## Abstract

**Background:**

To summarize the safety and effectiveness of high flow extracranial to intracranial saphenous vein bypass grafting in the treatment of complex intracranial aneurysms.

**Methods:**

The data of complex intracranial aneurysms patients for high flow extracranial to intracranial saphenous vein bypass grafting from January 2008 to January 2020 were retrospectively collected and analyzed. Eighty-two patients (31 men and 51 women) with 89 aneurysms underwent 82 saphenous vein bypass grafts followed by immediate parent vessel occlusion. The aneurysm was located at the internal carotid artery, middle cerebral artery, and basilar artery in 75, 11, and 3 cases, respectively.

**Results:**

The patency rate of bypass grafting was 100, 100, 96.3 and 92.4% on intraoperation, on the first postoperative day, at discharge and 6 months follow-up, respectively. At discharge and 6 months follow-up, 3 and 6 patients had graft occlusions. The main postoperative complications were transient hemiparesis and hemianopsia. 3 patients died due to bypass complications and poor physical condition.

**Conclusions:**

High flow extracranial to intracranial saphenous vein bypass grafting is safe and effective in the treatment of complex intracranial aneurysms and the saphenous vein can meet the requirements of brain blood supply. A high rate of graft patency and adequate cerebral blood flow can be achieved.

**Highlights:**

A single-centre long-term retrospective study was conducted to assess the safety and effectiveness of high flow EC-IC saphenous vein bypass grafting in the treatment of complex intracranial aneurysms. The data of 82 patients from January 2008 to January 2020 were retrospectively collected and analysed.

We found the patency rate of bypass grafting was 100, 100, 96.3 and 92.4% on intraoperation, on the first postoperative day, at discharge and 6 months follow-up, respectively. At discharge and 6 months follow-up, 3 and 6 patients had graft occlusions.

Finally, we conclude that high flow extracranial to intracranial saphenous vein bypass grafting is safe and effective in the treatment of complex intracranial aneurysms and the selected blood supply vessels can meet the requirements of blood supply.

As far as we know, this study is one of the maximum number of cases in the treatment of complex intracranial aneurysms with saphenous vein bypass.

## Background

Intracranial aneurysms, as a common cerebrovascular disease in neurosurgery, when treatment is indicated, are either treated by microsurgery or endovascular techniques. Both treatment options include several techniques and strategies [1]. However, some complex aneurysms, as defined by the aneurysm complexity score [2, 3], are not amenable to straightforward clip-reconstruction techniques or endovascular treatment. An individual approach needs to be suited then to these aneurysms, for which extracranial to intracranial (EC-IC) bypass option might be beneficial.

In 1969, Yasargil [4] performed and reported the first EC-IC bypass with ligation of the middle cerebral artery (MCA) in the successful treatment of a complex cerebral aneurysm. Since then, EC-IC bypass has remained important for the treatment of complex intracranial aneurysms. In particular, high flow EC-IC bypass (radial artery or saphenous vein) is more widely used in the treatment of complex intracranial aneurysms. In 1971, Lougheed et al. [5] first described saphenous vein as a conduit for EC-IC bypass.

In this study, the safety and effectiveness of high flow EC-IC saphenous vein bypass grafting for the treatment of complex intracranial aneurysms is illustrated in a single-center retrospective cohort of patients with long-term follow-up.

## Methods

### Patient series

In this study, the data of complex intracranial aneurysm patients for high flow EC-IC saphenous vein bypass grafting from January 2008 to January 2020 were retrospectively collected and analyzed. Basic demographics, aneurysm location social, preoperative clinical presentation, surgical outcomes, complications and follow-up information were recorded.

The choice of surgical technique was made by the responsible consultant. The Institutional Review Board (IRB) of Zhongnan Hospital of Wuhan University approved this study. The IRB waived the need for written consent. All patient data were anonymized and deidentified prior to analysis.

### Imaging evaluation and indications for bypass

**Fig. 1:**
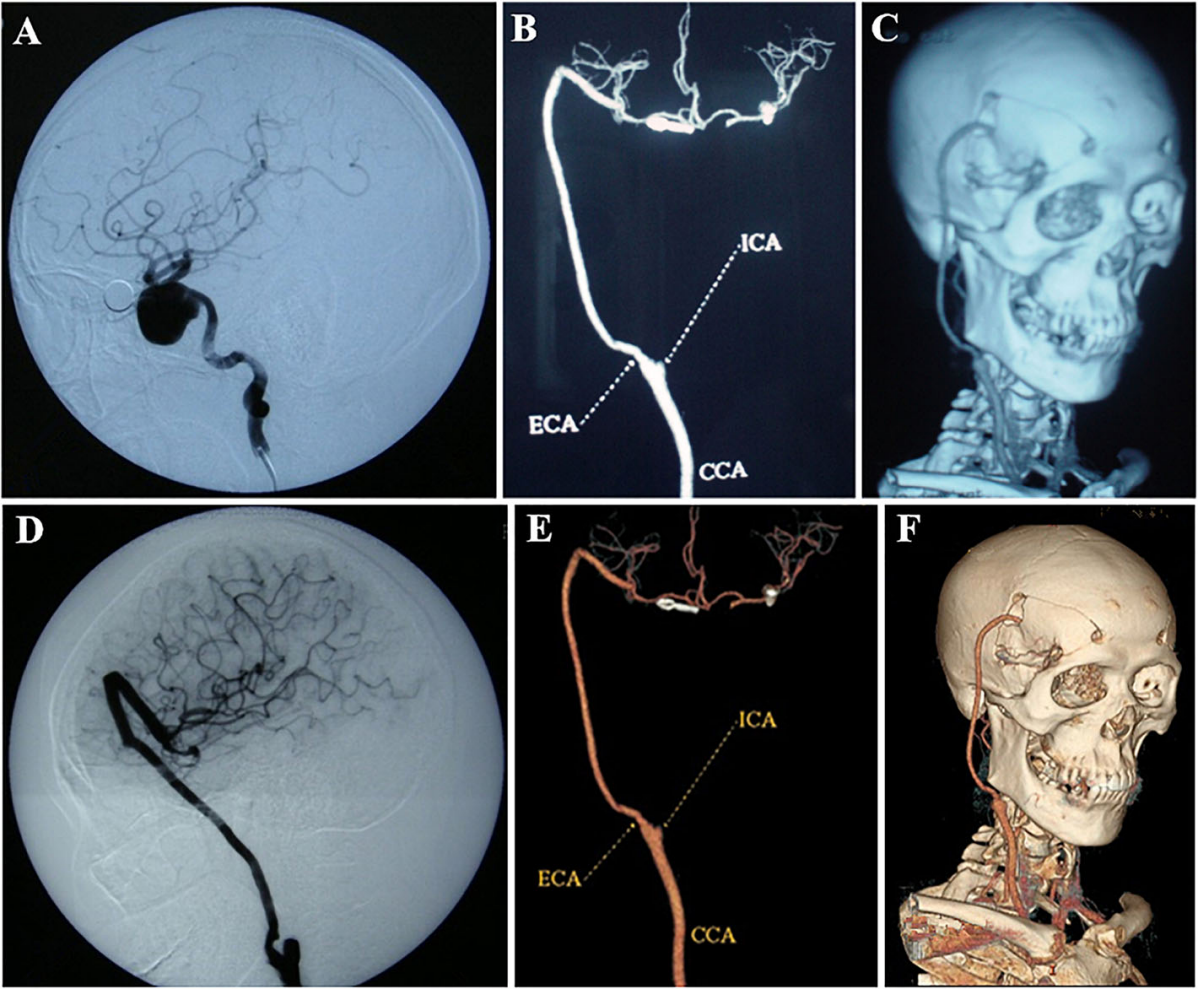
Preoperative, postoperative and follow-up imaging results. **A**: preoperative DSA can find a huge complex intracranial aneurysm in cavernous sinues; **B**, **C**: CTA results on the first day after operation and before discharge, we found that the aneurysm had disappeared and the bypass vessel was unobstructed.; **D**, **E**, **F**: DSA results at 6 months follow-up showed good

Digital subtraction angiography (DSA, Fig. 1A) and computed tomography angiography (CTA) were performed to evaluate aneurysms. Balloon test occlusion (BTO) is performed (as described by Elias AE, et al. [6]) to assess whether an artery can be temporarily or permanently blocked without significantly affecting brain blood flow. Bilateral computed tomography venography (CTV) was used to evaluate the diameter and branches of the saphenous vein. All patients were performed BTO and DSA or CTA. The results of BTO and DSA or CTA together determine the mode of operation.

### Surgical technique

**Fig. 2:**
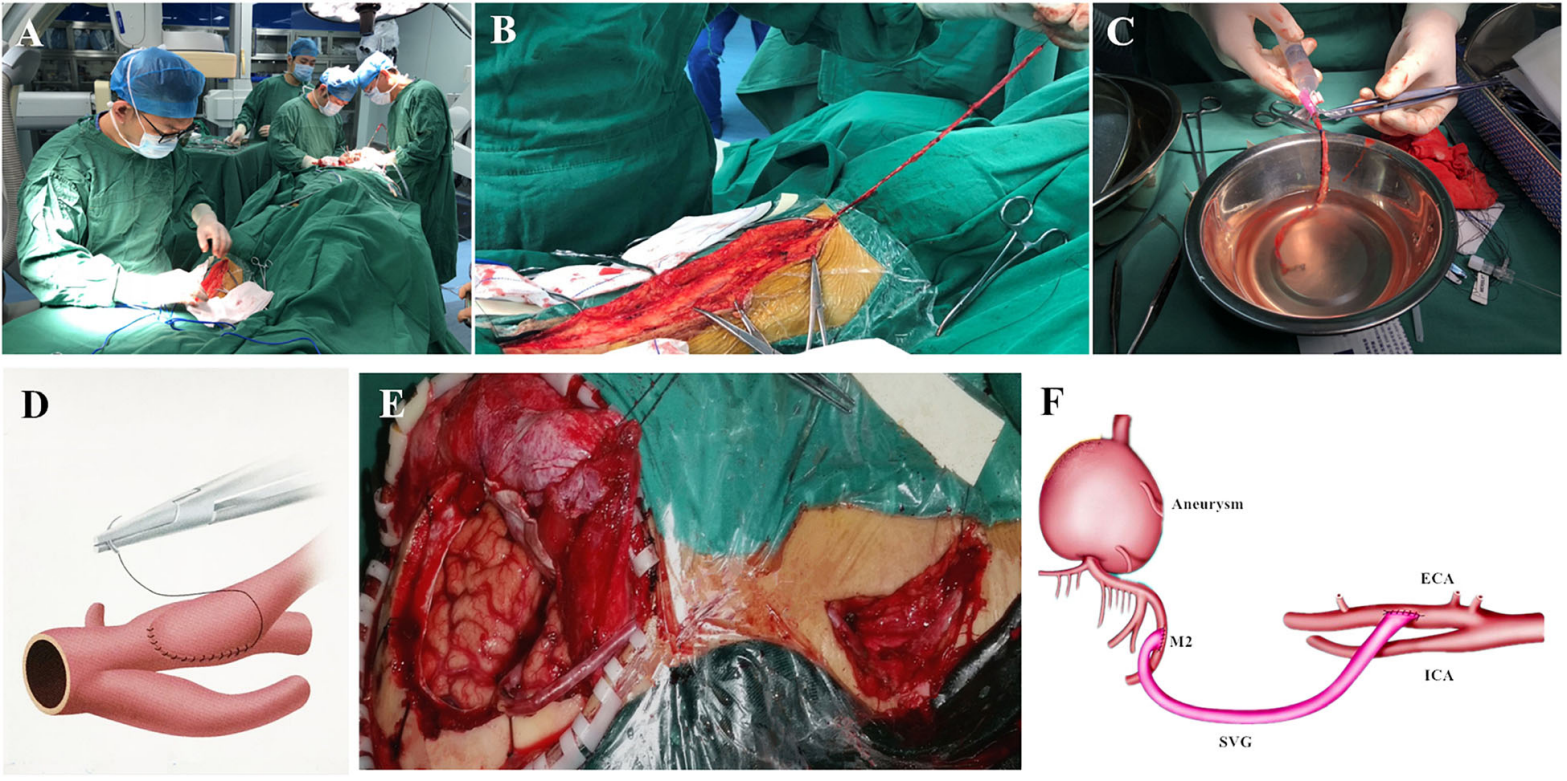
Operation process. **A**: The operation was performed by two groups of neurosurgeons. **B**: Operation to obtain great saphenous vein; **C**: The saphenous vein was flushed with 25 U/ml heparin saline; **D**: Conceptual image of vascular suture; The picture was painted by Jibo Zhang using Adobe Photoshop 2020. **E**: At the end of the suture, the blood vessels were checked to be unobstructed without blood leakage; **F**: Conceptual image of final effect. The picture was painted by Jibo Zhang using Adobe Photoshop 2020

The surgical technique of EC-IC bypass was described previously [7]. The operation was performed by two groups of neurosurgeons (Fig. 2A). One group of neurosurgeons performed frontotemporal (or pterional) craniotomy to expose the upper and lower branches of M2 segment of MCA, and then the cervical carotid artery. In the other group, the saphenous vein (guided by ultrasound machine, from the inside of the ankle to medial inferior of the knee joint, Fig. 2B) was taken through the incision of the lower limbs. After the saphenous vein was removed, it was flushed with 25 U/ml heparin saline (Fig. 2C). Before the suture of blood vessels, heparinization was carried out according to 1 mg / kg heparin sodium. First, the suture of saphenous vein and MCA was performed (Fig. 2D) with 8–0 or 9–0 monofilament nylon sutures, which should be completed within 30 min. The saphenous vein was anastomosed with the external carotid artery through the cervical subcutaneous tunnel. At the end of the suture, DSA was used to check the vascular patency and without leakage. Protamine (1 mg/kg) was injected intravenously to neutralize heparin. Finally, the aneurysms were isolated or clipped at the proximal end of the parent artery (Fig. 2E), and the aneurysmal content was evacuated after the isolation. After the operation, heparin sodium was used for DVT prophylaxis, and aspirin (100 mg/day) was taken at the 3rd day after operation. The patency of blood vessels was observed by the pulsation of subcutaneous blood vessels. CTA was performed on the first day after operation and before discharge to evaluate the general condition of arteries (Fig. 1B and C).

### Follow up

All patients were followed up by telephone at 1 month, 3 months and 6 months after operation to inquire about the symptoms and the pulsation of subcutaneous blood vessels. CTA or DSA should be performed at 6 months post-operation (Fig. 1E and F), after that CTA should be performed when indicated.

## Results

### Baseline demographics

In 12 years, 82 patients (31 men and 51 women) with 89 aneurysms underwent 82 saphenous vein bypass grafts followed by immediate parent vessel occlusion. The aneurysm was located at the internal carotid artery, middle cerebral artery, and basilar artery in 75, 11, and 3 cases, respectively. The average diameter of aneurysm was 32.3 (24–52) mm. The main preoperative clinical presentation were nervous system symptoms. The above information can be found in Table 1.

**Table 1:** Baseline demographics

	No. (%/Range)
Demographic	
No. of patients	82
Male	31
Female	51
Age (yr)	50.7 (29–63)
No. of aneurysms	89
Average diameter of aneurysm (mm)	32.3 (24–52)
Aneurysm location	
Internal carotid artery	75 (84.2%)
*Cavernous sinus*	61
*Ophthalmic*	14
*Posterior communicating artery*	3
Middle cerebral artery	11 (12.4%)
Basilar artery	3 (3.4%)
Preoperative clinical presentation	
Mass effect	59 (72.0%)
*With cranial neuropathy*	48
*With hemiparesis*	11
Headache and/or dizziness	27 (32.9%)
Vomiting	12 (14.6%)
Subarachnoid hemorrhage	19 (23.2%)
*Hunt-Hess grade I*	6
*Hunt-Hess grade II*	13
Seizure	2 (2.4%)

### Graft patency

The patency rate of bypass grafting, as confirmed by CTA or DSA, was 100, 100, 96.3 and 92.4% on intraoperation, on the first postoperative day, at discharge and 6 months follow-up, respectively. At discharge and 6 months follow-up, 3 and 6 patients had graft occlusions (Table 2). We thought thrombosis of perforating arteries caused by altered blood flow hemodynamics after parent vessel occlusion may be a continuing source of complications.

**Table 2:** Surgical outcomes, complications and follow-up

	No. (%)
Total Patients/ Bypasses	82/82
Patency rate of bypass grafts	
Intraoperation	100%
First postoperative day	100%
Discharge	79/82 (96.3%)
6 months follow-up	73/79 (92.4%)
Complications	
Transient hemiparesis	6/82 (7.3%)
Hemianopsia	2/82 (2.4%)
Death	3/82 (3.7%)
Modified Rankin Score at 6 months	
MRS 0–2	64/79 (81.0%)
MRS 3	7/79 (8.9%)
MRS 4,5	6/79 (7.6%)
MRS 6	2/79 (2.5%)

### Complications

The main postoperative complications were transient hemiparesis (6/82 (7.3%)) and hemianopsia (2/82 (2.4%)). For the 6 patients with transient hemiplegia, the postoperative muscle strength was grade II-III and recovered to IV-V grade at 7–10 days later, small infarction in basal ganglia area was found by CT. 3 patients died due to bypass complications (1 patient) and poor physical condition (2 patients) (Table 2).

### Follow-up

The patients were followed up for 6 months. The original symptoms of headache, dizziness and ptosis disappeared within 3 months after operation, and a small number of patients still had diplopia. CTA showed graft occlusions in 3 patients at 6 months after operation. The Modified Rankin Score at 6 months was 0–3 in most patients (71/79) (Table 2).

## Discussion

For surgical clipping or endovascular coiling, complex intracranial aneurysms are unique and complex challenges. High flow EC-IC bypass grafting can solve this problem well [8–10]. As far as we know, this study is one of the highest number of cases in the treatment of complex intracranial aneurysms with saphenous vein bypass. At present, saphenous vein and radial artery are mainly used in EC-IC bypass grafting. Some studies have shown that the radial artery is a better choice, because its intima is complete and consistent, the wall is thick, it is easy to suture and not easy to form thrombus in the intima; the diameter of the vessel is similar to the MCA, and the hemodynamic change is not significant. But the biggest disadvantage of radial artery is prone to spasm, which leads to failure of operation. However, we think the advantage of saphenous vein is that its diameter is relatively larger, it is not easy to have vasospasm, and it can ensure enough blood flow, it is relatively convenient and simple to obtain blood vessels, and it can ensure enough blood vessel length.

We got considerable results in this study, the patency rate of bypass grafting was 100, 100, 96.3 and 92.4% on intraoperation, on the first postoperative day, at discharge and 6 months follow-up, respectively. The results of this study are similar to those of other previous studies [8–12]. So, we can realize the safety and effectiveness of saphenous vein bypass grafting in the treatment of complex intracranial aneurysms. But there is also a certain failure rate. At discharge and 6 months follow-up, 3 and 6 patients had graft occlusions. We thought thrombosis of perforating arteries caused by altered blood flow hemodynamics after parent vessel occlusion may be a continuing source of complications [10]. Therefore, it is very important to evaluate the hemodynamics of the brain and control the indications of the operation.

The indications for high flow EC-IC saphenous vein bypass grafting were these: large and complex aneurysms, aneurysms with a large and complex neck not suitable for clip reconstruction, blister aneurysms, dissecting aneurysms, dissecting aneurysms, aneurysms with an origin of branch vessel(s) from the aneurysm sac, calcification or atherosclerotic changes of the aneurysm neck, extensive thrombosis inside the aneurysm, and recurrent aneurysms that had failed endovascular or microsurgical treatment [13].

About ruptured and unruptured aneurysm, we think whether the aneurysm is ruptured or not, we should carry out surgical treatment as soon as possible as soon as the patient’s vital signs are stable. Especially, for the huge and complex ones. And SAH or intracranial hematoma can also be cleared by surgical treatment.

In the past, there has always been a question, when to isolate the aneurysm? Is it isolated directly during the operation or after CTA or DSA confirms that the blood vessels are unobstructed? However, the emergence of hybrid operating room, which has both interventional radiology and surgical equipment, has greatly solved this problem. After microsurgery, DSA was performed directly (in the same operating room) to evaluate whether the blood vessels were unobstructed or not as soon as possible. Therefore, in the future, we encourage the operation in hybrid operating room. It makes the intraoperative vascular evaluation more effective. After the establishment of the hybrid operating room in our center in 2017, the operation is carried out in it. In addition to the intraoperative DSA can be used to evaluate the patency of blood vessels, the intraoperative fluorescein angiography and ultrasound can also be used. In fluorescein angiography, indocyanine green was used to show the blood vessels under the microscope. Ultrasound uses a probe to monitor the patency of blood vessels. Actually, all three have similar functions.

## Conclusions

High flow extracranial to intracranial saphenous vein bypass grafting is safe and effective in the treatment of complex intracranial aneurysms and the saphenous vein can meet the requirements of blood supply. A high rate of graft patency and adequate cerebral blood flow can be achieved.

## Data Availability

The data that support the findings of this study are available on request from the corresponding author.

## Abbreviations

BTO: Balloon Test Occlusion
CTA: Computed Tomography Angiography
CTV: Computed Tomography venography
DSA: Digital Subtraction Angiography
EC-IC: Extracranial-to-Intracranial
MCA: Middle Cerebral Artery
